# Towards Stronger Gambling Regulation in Ghana: A Critical Frame Analysis of Ghana Gaming Act

**DOI:** 10.1002/puh2.70227

**Published:** 2026-04-09

**Authors:** Emmanuel Badu

**Affiliations:** ^1^ Collaboration for Evidence, Research and Impact in Public Health School of Population Health Curtin University Perth Western Australia Australia; ^2^ School of Population Health Curtin University Perth Western Australia Australia

**Keywords:** commercial determinants, gambling, gambling regulation, legal determinants, public health approach

## Abstract

Ghana enacted the Gaming Act (Act 721) in 2006 to formalise and liberalise the gambling market. Ghana's gambling landscape has evolved substantially since the enactment of the Act. The gambling market has expanded, and gambling has become a common pastime activity in Ghana. This article examines how Ghana's legal framework may be contributing to the rise in gambling behaviour and related harms in Ghana. Specifically, this study explores how gambling problems and solutions are framed and how these influence commercial gambling industry activity as a vector of gambling behaviour and harms. The analysis identified consumer protection and elimination of illegal elements as the main frames associated with the ‘problem’. The solutions provided in the Act were mostly aligned with two frames: consumer protection and liberalisation or formalisation of the market. These frames are typically aligned with the responsible gambling framing and centre on the individual without adequate levers for effective public health response to gambling and gambling‐related harms. Ghana's Gaming Act is no longer fit for purpose. There is an urgent need to review and strengthen the Act with an explicit focus on public health principles and approaches to protect the citizens from these harmful products and industries.

## Graphical abstract

Key findings

The Act is not fit for purpose; it needs urgent review with an explicit focus on public health principles to better protect citizens against gambling harms. 2

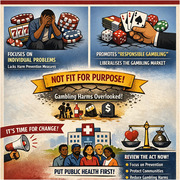

## 1 Introduction

In 2006, Ghana enacted the Gaming Act 2006 (Act 721) (hereon referred to as the Act) ‘to revise and consolidate all the laws relating to casino and other gaming activities other than lottery and also provided for ancillary matters concerning the gaming industry’ [1]. The Act legalised and liberalised Ghana's gaming (gambling) market in line with global trends at the time [2, 3]. The Act has three parts: Part I relates to gaming and betting, including establishing the Gaming Commission with the mandate ‘to regulate, control, monitor and supervise the operation of games of chance in the country’ [1]. Part II deals with gaming machines, and Part III relates to regulation of casinos.

The Act introduced major reforms to gambling regulation in Ghana, such as the establishment of a dedicated regulatory entity. Prior to its enactment, the regulatory framework was fragmented, with separate laws governing various aspects of gambling activities. Since the Act's passage in 2006, however, Ghana has not implemented further regulatory reforms, despite a marked increase in gambling participation and associated harms. The aim of this article is to examine how Ghana's legal framework may be contributing to the rise in gambling behaviour and related harms in Ghana. Specifically, this study explores how gambling problems and solutions are framed and how these influence commercial gambling industry activity as a vector of gambling behaviour and harms.

Ghana's gambling landscape has evolved substantially since the enactment of the Act. Currently there are 28 licenced sports betting operators, 23 casino operators and 16 online casino operators (many are international sports betting operators) [4]. Gambling, especially sports betting, has been popularised and normalised, becoming a common pastime activity in Ghana [5]. The normalisation of gambling, especially sports betting, has been associated with an increasing trend in gambling‐related harm, including financial stress, poor mental health and wellbeing, poor academic performance and challenges with personal and social life, including family breakdown and crime [5, 6, 7].

Ghana's gambling market is projected to reach about $915 million revenue in 2025, with online casino and sports betting markets experiencing significant growth [8]. The ubiquity of online gambling opportunities, mixed with a high rate of youth unemployment, and a growing middle class with increasing disposable incomes are driving the online sports betting and online casino markets [8].

The expansion of the gambling industry is not unique to Ghana. A similar trend has been observed in other sub‐Saharan African countries [9, 10]. With high income countries tightening their regulations of the gambling industry, many big gambling industries have moved to new frontiers to expand their markets and increase profits for their shareholders [11]. The prevailing weak regulatory regimes in countries, such as Ghana, have meant the industry has unfettered access to drive gambling behaviour [12, 13]. Although the evidence regarding gambling industry activities and influence on gambling behaviour and harms in sub‐Saharan Africa continues to build [14, 15, 16], it is quite telling that the industry has used similar tactics from the playbook of other harmful industries such as tobacco [17], alcohol [18, 19] and food [20, 21, 22] to expand rapidly within a very short timeframe.

Gambling regulation is one area where the conflict between neoliberal forces of market liberalisation, industry profits, government revenue generation for debt servicing and the state's duty to protect public and social interests become apparent. Law, such as the Gaming Act of Ghana, is a determinant of health as ‘it exerts a powerful influence on health by structuring, perpetuating and mediating the risk factors and underlying conditions known as the social determinants of health’ [23]. Law establishes the rules and frameworks that shape economic interactions and social outcomes [23]. It can ensure safe and healthier environments. Conversely, law can harm people, particularly the most vulnerable in society, when poorly designed, implemented or enforced [23]. In the case of gambling regulation, law legitimises commercial gambling by constructing, justifying and authorising gambling operators and their activities [24]. For example, gambling operators in Ghana use regulatory approval by the Gaming Commission to signal legitimacy of their advertisements [25].

Globally, gambling regulation remains heavily focused on individual‐level factors [26] despite calls by scholars for a public health approach to preventing and minimising gambling harms [11, 27, 28]. Governments have created laws that stimulate market growth and engender competitive environments to increase their revenues while encouraging the industry to ensure ‘responsible adults’ can enjoy gambling responsibly free from criminal elements [2, 26, 29]. This is known as the responsible gambling approach [2]. Responsible gambling focuses on individual agency, believing the individual is ultimately responsible for their gambling behaviour [11, 30]. It is based on the notion of individual freedom and informed choice, and it shifts the responsibility for addressing gambling‐related harms from governments and commercial gambling industry to consumers by emphasising minimal interventions, education, self‐management and fewer restrictions [2]. Responsible gambling framework has been shown to be ineffective in comprehensively addressing industry's predatory practices [28, 31] and has contributed to the situation where countries like Ghana continue to ignore the contribution of the industry in the production of harm through their products and pervasive marketing tactics [11].

## 2 Methods

A critical frame analysis was used to identify and examine how gambling and gambling harms are framed within Ghana's gambling legislation. A critical frame analysis interprets how meanings are constructed and how these in turn shape government action [32]. This approach is concerned with the use of symbolic tools or interpretation schemes or frames to structure or give meaning to how policy actors define and propose solutions to problems, whether implicitly or explicitly [33, 34]. The Act is a construction of reality; it is the framework that guides how gambling behaviour and harms are defined and regulated in Ghana.

Drawing on this approach [32, 33] and the work of [26] a series of questions was adapted to inform the analysis. These questions broadly sought to understand the nature of the problem (what was wrong), who and what was responsible for the problem and the proposed solutions or actions (prognosis). These specific questions were used:

*What is wrong (nature of the problem/diagnosis)*: How is the nature of gambling addiction or gambling‐related harms identified? Is gambling framed as safe for the majority of players? Are harms understood as only individual harms? Or are social and population‐level harms also considered?

*Who or what is responsible for the problem*: What is identified as a key cause or risk factor of gambling addiction or gambling‐related harms? Illegal or unregulated markets? Product availability? Product design? Marketing promotions and advertising?

*Proposed solutions or actions (Prognosis)*: Is the responsible gambling principle explicitly invoked? Who is considered responsible for harm prevention, and in what way? What policy measures are proposed to tackle gambling harms? Are these measures focused on addressing individual or structural causes of gambling harms? Do they target the whole population? Or only vulnerable groups (e.g., underage bettors and at‐risk players)?

The analysis of the proposed solutions (prognosis) focused on key principles of public health approaches or responses to preventing gambling harm [28]. A list of gambling harm prevention strategies that align with individual and public health responses was used [26].

## 3 Results

The analysis demonstrates the Act frames gambling as a game of chance that could be enjoyed by responsible adults and that the government's role is to ensure that individuals are protected from fraudulent or unscrupulous operators (see Table 1). The findings from each sub‐theme are presented in the following sections.

**TABLE 1:** Summary of findings from critical frame analysis of the Act.

Critical frame dimension	Category/Questions	Yes (✓)/No (x)
Nature of the problem (diagnosis)	Are harms understood as only individual harms (consumer protection)?	✓
	Gambling addiction referred to directly?	x
	Are social and population‐level harms also considered?	x
Who or what is responsible for the problem?	Product design	x
	Gambling marketing (including advertisement)	x
	Unregulated/Illegal gambling/Black market/Grey market	✓
	Availability of gambling	x
Solutions or actions (prognosis)	Ban on minors/youth gambling	✓
	Limiting gambling outside gambling venues	✓
	Licence required and must be displayed	✓
	(Self)‐exclusion	x
	Restricting marketing	x
	Restricting location of venues	x
	Limiting product design	x
	Limiting number of venues	x
	Funding for prevention	x
	Limiting gambling venue hours	x

### 3.1 Nature of the Problem (Diagnosis)

The analysis identified consumer protection and elimination of illegal elements as the main frames associated with the ‘problem’. Although the Act does not explicitly define the nature of the problem, it implicitly alludes to the presence of criminal or illegal elements in an informal gambling market driving gambling harms such as non‐payment of winnings and exploitation of consumers. This is apparent in the main rationale underpinning the types of solutions that were prioritised to address the problem as highlighted in the Act and below:

Section 14(1)(c). An operator must meet the minimum capital requirement: ‘has the required minimum stated capital under Section 22 and has agreed to maintain the amount of cash or cash equivalents determined by the Commission’.

Section 14(1)(d) ‘has submitted a criminal clearance certificate in respect of all directors of the company’.

Section 14(2). ‘The Commission shall not grant a licence if any of the directors of the applicant company has (a) been adjudged insolvent or bankrupt and has not been discharged, (c) been convicted by a court or tribunal of an offence involving fraud or dishonesty and an appeal against conviction has not been brought or having been brought was withdrawn or dismissed’.

Section 20(b). The Commission may revoke a licence where an ex‐convict whose conviction is less than ten years is appointed as a director of the company.

Section 20(c). A Company is Unable to Maintain the Amount of Cash or Cash Equivalents Required.

The analysis further indicated that references to gambling addiction or social and population‐level gambling‐related harm were entirely absent from the Act. The legislation did not explicitly consider or address the prevention of, and protection from, gambling‐related harms as part of its objectives.

### 3.2 Who or What Is Responsible for the Problem?

The cause of gambling harms was not explicitly articulated or elaborated in the Act. Analysis of the text shows that prior to the enactment of the law, the gaming market was largely unregulated. It was mostly informal market, dominated by unlicenced, illegal or pseudo‐operators who were exploiting vulnerable people. The introduction of the Act sanitised the gambling environment, protected consumers and ensured that formal systems were introduced to regulate and monitor the operations of gambling operators.

### 3.3 Solutions or Actions (Prognosis)

The solutions provided in the Act were mostly aligned with two frames: consumer protection and liberalisation or formalisation of the market for economic benefits to consumers, the industry and government.

#### 3.3.1 Liberalising or Formalising the Market

The Act prioritised formalisation and liberalisation of Ghana's gambling market. For example, the Act uses simplified rules to encourage market growth while also protecting Ghanaian interest Section 14(1)(g): ‘… is partly or wholly Ghanaian owned’. In furtherance to formalising and regulating the market, the Act establishes the Ghana Gaming Commission in Section 2. It further requires all operators of games of chance to be licenced by the Gaming Commission's Board (Section 13(b)) and to renew their licences annually (Section 21(1)). All gaming machines must be licenced (Section 43(1)). Gaming machines imported must be explicitly approved by the Gaming Commission Board (Section 41(1)). All casinos must be licenced (Section 60(1)): ‘a notice that a casino is licenced shall be prominently displayed’. Section 60(2): ‘The notice shall include reference to the games of chance authorised under the licence, the maximum percentage of commission to be deducted in play and the odds to be paid as appropriate’.

It also sanitised gaming in public Section 43(b) ‘A person who plays a game or pretended game of chance at a table or with a card, coin, token or other article used as an instrument or means of betting or gaming which is not approved for betting and gaming by the Board, commits an offence and is liable on summary conviction to a fine … or a term of imprisonment…’. Gaming and betting houses must receive approval of the Board. A person found in an unapproved gaming and betting house commits an offence. It restricts the use of the word ‘casino’ (Section 61). A person or body of persons shall not use the word ‘casino’ in conjunction or as part of that person's or the body's name without the prior written consent of the Board.

#### 3.3.2 Consumer Protection

The Act sets out terms and guidance on how individuals could engage in gaming (gamble) responsibly. For example, it provides guidance on cheating in gaming (Section 37), gaming and betting agreements (Section 38), and loans for gaming or betting (Section 40). To protect vulnerable people from the perceived harms, the Act prohibits children (less than 18years old) from gaming or entering a place where gaming is operated (Section 48). It also protects consumers against defaults in payment of their wins, requiring licence holders to have minimum capital requirements (Section 22) and minimum bankroll requirements (Section 23). Section 24 deals with penalties for non‐compliance with bankroll requirements. Section 25 deals with dishonoured cheques.

## 4 Discussion

The results show that the Act provides simplified rules to formalise the gambling market, enable more private sector participation, protect consumers from unscrupulous providers, provide customers with confidence in the industry and allow the industry to self‐regulate. These are typically aligned with the responsible gambling framing and are favoured by commercial gambling industry [2, 11, 26, 35]. It does not provide adequate levers for effective public health response.

The adopted solutions in the Act are mostly centred around the individual, reflecting how gambling was identified and defined as a problem. Hoefer [36] points out the ambiguous and political nature of problem definition and adoption of solutions, as different actors may define the same situation differently and offer varied solutions. What gets enacted and implemented thus depends on definitions and solutions that are acceptable and appealing to decision‐makers, the national mood, international debates and a political will to act [36]. The Act's does not allow for structural efforts at prevention and minimisation of harm across the entire population [37]. It mostly offers protections for the ‘money’ or ‘transaction’ involved between three main actors—the consumer, gambling operators and the government. It protects the wins of the consumer, enables more profits for operators and aims to increase revenues for the government through licences and registration fees [38]. The Act does not explicitly aim to prevent or minimise the harms of gambling. Harms associated with gambling such as mental health concerns, violence, family breakdown, educational impacts, suicidality and physical health [5, 39] are not within the purview of the Act or the Commission.

The Act is silent on the design of gambling products and platforms. Ghana's regulatory body has no mandate or oversight responsibility for the type, features or nature of games, their design and how these are implemented. The industry self‐regulates through industry‐generated codes of responsible conduct. Self‐regulation has been shown to be ineffective, with evidence from Finland showing gambling operators use self‐regulation to inhibit government regulation efforts [35]. Commercial gambling industry strongly influences how they are regulated, often seeking to shape and advocating for self‐regulation to ward off government action [24]. However, the international literature shows gambling products are not mere products; they are harmful products by design [40], with those characterised by fast, intense and continuous play such as internet gambling, leading to increased risk of harm [41, 42]. The lack of specific regulation on gambling product design features in the Act limits the Commission's ability to monitor and enforce them. Operators have the unrestrained ability to evolve new products without recourse to any safety or consumer protection standards. The call for regulations governing gambling product and platform design and promotion aligns with global call by gambling scholars [11].

The Act's valorisation of individualised behavioural solutions is driven by neoliberal ideologies of market liberalisation, competition and profits [43, 44]. These have contributed to the rise in gambling opportunities in Ghana as operators compete and aggressively market their products, ensuring widespread availability and accessibility [5, 10, 16]. The rapid expansion of the gambling industry in Ghana has been facilitated by the regulatory framework which has streamlined application for licence and registration processes, enabling new operators and encouraging competition. International research has shown governments have sought to stimulate economic growth by enabling commercial gambling expansion, creating gambling‐related jobs and increasing revenues without increasing taxes [11, 27, 45]. Protecting the public from the harms of gambling seems to be of less priority to the government on the face of the Act. The persistent inaction by successive governments to substantially review or amend the Act over the past two decades, despite the rapid evolution of gambling products, raises critical concerns. This inertia may reflect a tacit complicity or an overarching prioritisation of revenue generation over the regulation of emerging gambling‐related risks. The calls on the Ghana government to act [10, 16] should be prioritised, including a commitment to a comprehensive review of the Act.

The analysis shows a new act is imperative with the explicit focus on prevention and protection of people from the harms associated with gambling [10, 46, 47]. As emphasised by The Lancet Public Health Commission on Gambling, public health concerns must take precedence over industry profits and governments’ revenue interests [11]. Efforts must be made to protect the new Act from the influence of the industry [28]. Evidence abounds on how the industry will continue to lobby for weaker regulations to advance their profits and shareholder interests [48]. As is the case for tobacco control regulation, the industry should have no role in designing regulations to prevent and protect people from the harms they produce or cause. The new Act must address widespread availability and accessibility of gambling products through restriction on locations of gambling venues, strengthen the Gaming Commission for effective oversight, strengthen gambling advertising regulations and improve monitoring and enforcement mechanisms.

## 5 Conclusion

Ghana's Gaming Act frames gambling as an individual problem with less focus on the prevention and minimisation of gambling‐related harms. The Act formalised and liberalised the gambling market using predominantly responsible gambling framing. Currently, the Act is not fit for purpose. The gambling market has expanded exponentially since the enactment of the Act and is driving gambling behaviour and harms in Ghana. There is an urgent need to review and strengthen the Act with an explicit focus on public health principles and approaches to protect the citizens from these harmful products and industries.

## Author Contributions

**Emmanuel Badu**: conceptualization, writing – original draft, methodology, writing – review and editing, visualization, formal analysis, investigation.

## Funding

The author has nothing to report.

## Ethics Statement

The author has nothing to report.

## Conflicts of Interest

The author declares no conflicts of interest.

## Data Availability

No new data were generated in support of this research.
